# GPS-Free Wireless Precise Positioning System for Automatic Flying and Landing Application of Shipborne Unmanned Aerial Vehicle

**DOI:** 10.3390/s24020550

**Published:** 2024-01-15

**Authors:** Tsu-Yu Lo, Je-Yao Chang, Tan-Zhi Wei, Pin-Yen Chen, Shih-Ping Huang, Wei-Ting Tsai, Chong-Yi Liou, Chun-Cheng Lin, Shau-Gang Mao

**Affiliations:** 1 Graduate Institute of Commutation Engineering, National Taiwan University, Taipei 106, Taiwan; 2 Department of Power Vehicle and Systems Engineering, Chung Cheng Institute of Technology, National Defense University, Taoyuan 335, Taiwan

**Keywords:** unmanned aerial vehicles, wireless positioning, ultra-wideband, autonomous flying and landing system

## Abstract

This research is dedicated to developing an automatic landing system for shipborne unmanned aerial vehicles (UAVs) based on wireless precise positioning technology. The application scenario is practical for specific challenging and complex environmental conditions, such as the Global Positioning System (GPS) being disabled during wartime. The primary objective is to establish a precise and real-time dynamic wireless positioning system, ensuring that the UAV can autonomously land on the shipborne platform without relying on GPS. This work addresses several key aspects, including the implementation of an ultra-wideband (UWB) circuit module with a specific antenna design and RF front-end chip to enhance wireless signal reception. These modules play a crucial role in achieving accurate positioning, mitigating the limitations caused by GPS inaccuracy, thereby enhancing the overall performance and reception range of the system. Additionally, the study develops a wireless positioning algorithm to validate the effectiveness of automatic landing on the shipborne platform. The platform’s wave vibration is considered to provide a realistic landing system for shipborne UAVs. The UWB modules are practically installed on the shipborne platform, and the UAV and the autonomous three-body vessel are tested simultaneously in the outdoor open water space to verify the functionality, precision, and adaptability of the proposed UAV landing system. Results demonstrate that the UAV can autonomously fly from 200 m, approach, and automatically land on the moving shipborne platform without relying on GPS.

## 1. Introduction

The necessity to maintain order at sea has driven advancements in maritime defense technology worldwide, particularly in island regions. Maritime patrols heavily rely on a diverse array of radar systems for effective target detection. Traditional reconnaissance missions, often conducted by manned aircraft, pose significant challenges, encompassing high costs and intricate operational complexities. Consequently, the use of unmanned aerial vehicles (UAVs) becomes a more effective solution to simplify the execution of reconnaissance and identification tasks [1].

The automatic flying and landing systems of UAVs typically rely on traditional infrared and GPS positioning methods. However, in the dynamic maritime environment, they are susceptible to environmental and signal interference, leading to potential damage to UAV equipment and landing failures [2].

Various positioning technologies are currently employed, including ultrasound [3], infrared [4], Wi-Fi [5], Bluetooth Low Energy (BLE) [6], laser [7], cameras [8], and ultra-wideband (UWB) [9,10,11]. Infrared sensors detect voltage or heat variations based on materials and wavelengths, while Wi-Fi utilizes signal strength or channel state information to establish positioning models [12,13]. BLE and Wi-Fi share a similar principle but require their own base station for signal provision, with precision falling short of Time-of-Flight (TOF) technology. Laser and ultrasound both use TOF for accurately measuring the distance to reflecting objects, with laser positioning requiring suitable variations in reflective objects in the environment, potentially leading to challenges in specific environments. Cameras can determine the depth and angle of target objects and extract features through image recognition, yet their applications are limited by light conditions and limited coverage angles, Table 1 provides a comprehensive overview of the advantages and disadvantages of each positioning technology.

UWB positioning technology exhibits notable advantages in maritime UAV positioning applications, offering high-precision positioning unaffected by environmental light and capable of measuring distances ranging from tens to hundreds of meters. In contrast, positioning technologies based on ultrasound, infrared, laser, and cameras encounter difficulties in achieving stable detection results due to the smaller size of the target UAVs and susceptibility to environmental light, rendering them unsuitable for maritime UAV positioning. Additionally, the precision of wireless positioning technologies such as Wi-Fi and Bluetooth reaches only the meter level, insufficient for precise UAV landings. UWB positioning technology boasts precision at the centimeter level, unaffected by environmental light, and capable of detecting distances ranging from tens to hundreds of meters [14]. Consequently, this project adopts UWB wireless positioning technology to assist in achieving the precise landing of UAVs.

Although there has been previous research on using UWB for UAV landing positioning [15,16], the differences and limitations in these studies are compared and discussed. The study [15] focuses on logistics transportation, integrating GPS modules, cameras, and UWB modules for accurate UAV positioning. However, this method may become less reliable in situations with unstable GPS signals, and cameras may not provide precise positioning in low-visibility conditions. It is also tailored to specific application scenarios, such as urban cargo transportation, where landing locations are typically at fixed horizontal planes. This differs from our current research, which aims to achieve UAV landing on a moving ship with a dynamic and vibration plane.

In the research carried out by [16], the UAV landings on a moving truck with different terrains like uneven roads, hills, and marine environments is proposed. The GPS module, Inertial Measurement Unit (IMU) module, and UWB module are integrated for wireless localization. However, due to the limited effective range of the UWB module, positioning without GPS reliance is only within 30 m. This implies strict usage constraints. Additionally, the User Datagram Protocol (UDP) transmission is used in this work to relay GPS and IMU data from the landing location to the UAV. This extra communication requirement might affect the system operation in cases of additional wireless channel interference.

Therefore, this article proposes a new type of GPS-free wireless precise positioning system, which extends the communication distance through UWB modules with RF front-end modules, and improves positioning accuracy through positioning algorithms to eliminate dependence on GPS modules. At the same time, integrating IMU into UWB communication can ensure that all information is transmitted through the UWB system. This innovation enables our system to enable autonomous UAV landing without the need for additional communication channels. Our goal is to achieve high-precision UAV landing in dynamic ocean environments without GPS signals.

This study proposes a UWB wireless precise positioning system for shipboard aircraft that operates without GPS assistance. The UWB module, including antenna and IC, are developed. In addition, the UAV automagical landing system is realized and verified by using dynamic vessel platform. The major contributions of this study are summarized as follows:

- Hardware: The UWB antenna and the corresponding module with ICs are designed, fabricated, and measured. A good agreement between simulation and measurement is obtained.
- Software: A positioning algorithm with optimal gradient descent method is developed and embedded into 32-bit MCU to localize the drone’s 3D coordinates.
- System integration: The application scenario of drone landing is established and the shipborne UAV automatic landing system is realized. Hence, the usefulness of our proposed technology in adapting to various complex environments is verified.

This paper is organized as follows. Section 2 provides an outline of the design of a UWB wireless precise positioning system without using GPS. Section 3 fabricates the antenna and measures the radiation characteristics. A simulation and experiment are conducted to prove the wireless positioning algorithm. The drone flight control system and the vessel are built as an experimental platform. The integration and verification of the wireless positioning system and ship-borne platform test is implemented in Section 4. The comparison of measured and simulated 3D wireless positioning results is discussed in Section 5. The conclusion is drawn in Section 6.

## 2. GPS-Free Wireless Precise Positioning System Design

### 2.1. UWB Module Design

#### 2.1.1. Antenna Design

In the design of UWB positioning systems, the layout and configuration of the antenna hardware play a crucial role. The performance of the antenna has a direct impact on positioning accuracy and coverage. To ensure optimal performance for UWB positioning applications, the antenna should possess key properties such as omnidirectionality and minimal changes in group delay with variations in angle [17].

**Figure 1:**
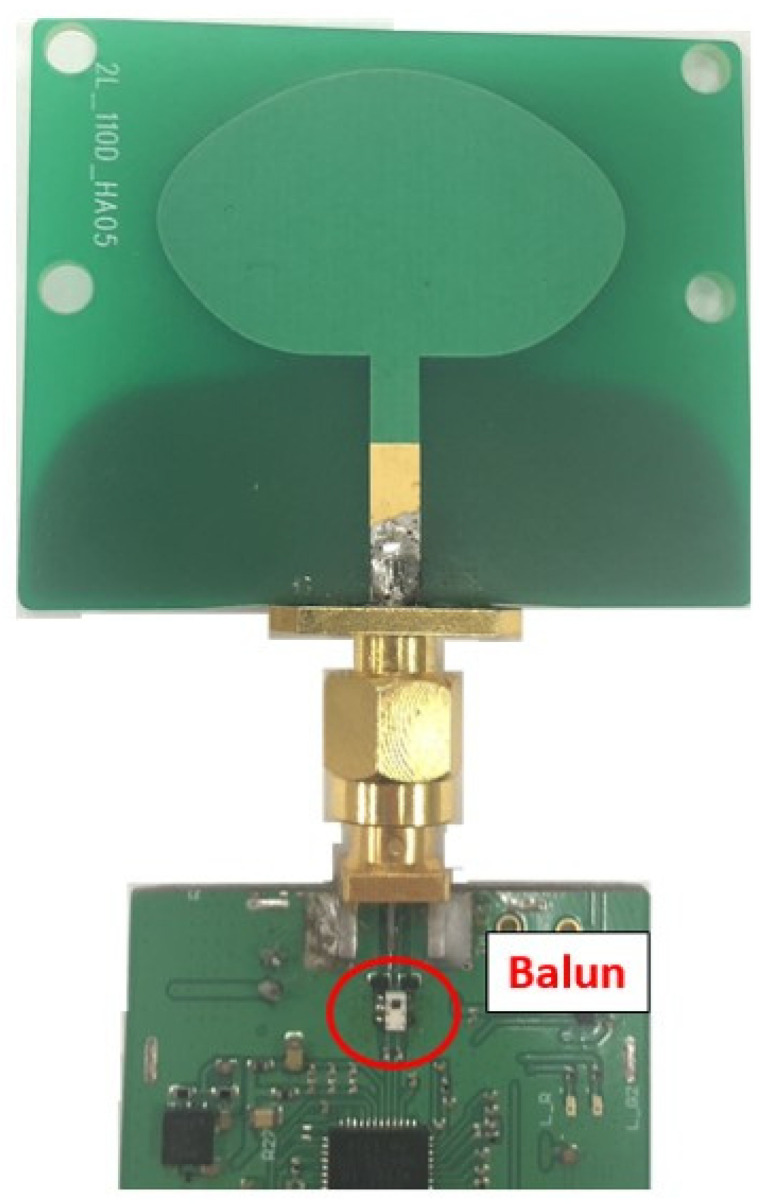
Balun structure (marked in red circle) for connecting single-end feed line with module.

Furthermore, as UWB chips typically transmit signals differentially, connecting them to a single-ended antenna necessitates the addition of a balun structure [18] for differential to single-ended conversion, as illustrated in Figure 1. This balun structure not only occupies space but also increases manufacturing costs and affects the antenna’s characteristics. To eliminate the need for a balun, the most effective connection method is direct differential input. Hence, the primary objective of this antenna design is to create a compact UWB antenna with differential input, considering the influence of the module, while maintaining desirable characteristics such as wide frequency coverage, omnidirectionality, and consistent group delay at various angles.

**Figure 2:**
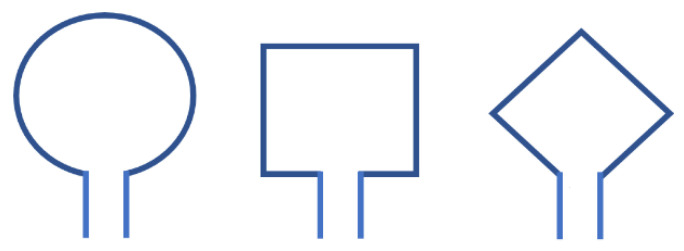
Ring antenna structures of different shapes.

In antenna design based on UWB positioning systems, broadband loop antennas exhibit some key advantages. The structure of a loop antenna, as shown in Figure 2, is usually a metal wire wound into a loop, which can be circular, square, rhombus, etc. Its characteristic lies in the circulation of a current along the axis, which can be equivalently represented as a magnetic dipole situated on that axis [19]. Compared with electric dipoles, magnetic dipole loop antennas are less affected by the human body and are therefore more suitable for use in wearable devices [20]. This means that in UWB positioning systems, especially when the application requires contact with the human body or is worn on the body, the choice of loop antenna is more appropriate.

**Figure 3:**
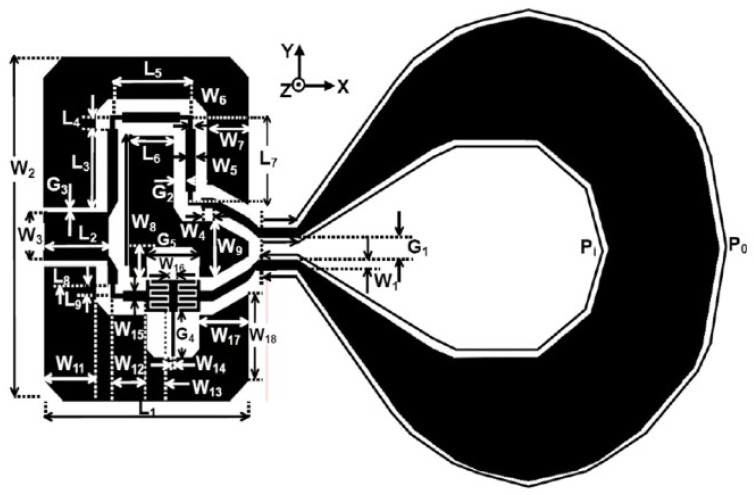
The tapered loop antenna.

For UWB positioning systems, although small loop antennas offer the advantages of simple structure and minimal human body interference, their bandwidth is insufficient. To increase the bandwidth, a common approach is to use a tapered loop antenna [21], as shown in Figure 3. The tapered design provides the antenna with different lengths of resonant current paths, thus enabling multiple resonant modes at different frequencies. 

**Figure 4:**
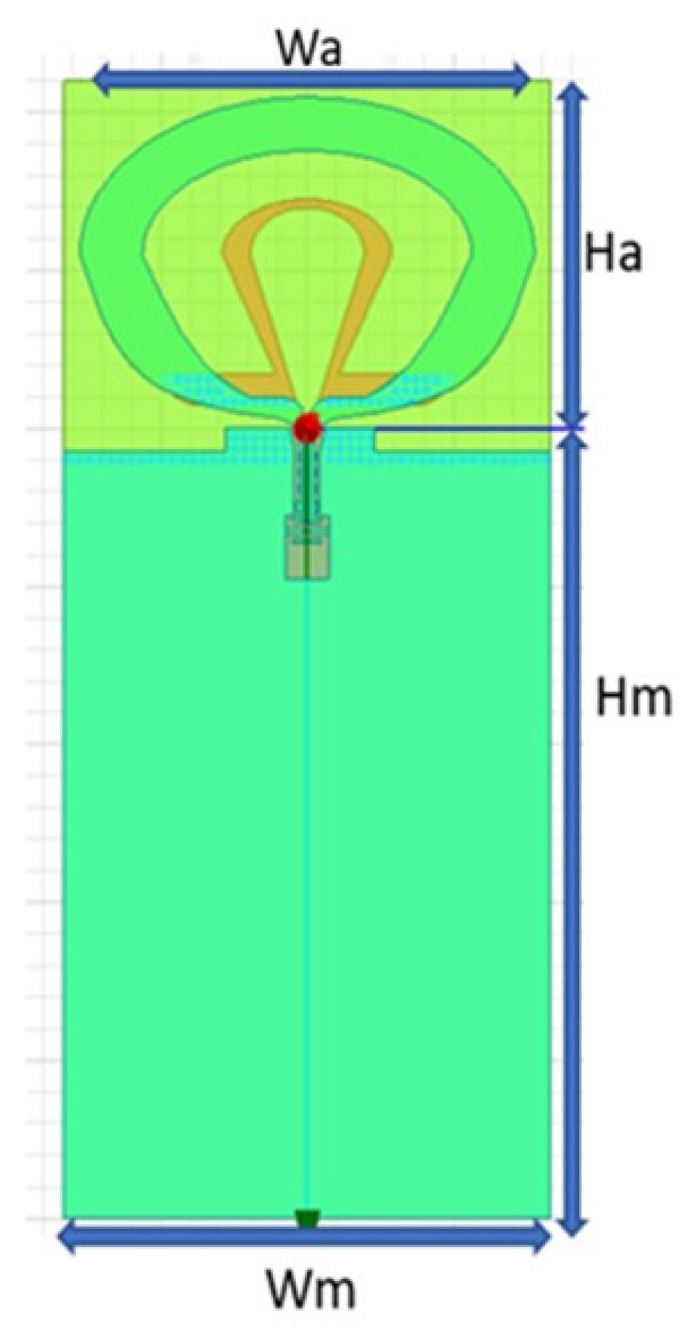
Double-ring antenna with module board.

This design introduces a dual-band dual-loop antenna for a UWB positioning system, depicted in Figure 4. To better align with practical application requirements, the design takes into account the connected rear module and employs a four-layer FR4 board for simulation. The overall dimensions of the antenna are 21 × 31 (Ha × Wa) mm, and the total size, including the module, is 71 × 33.6 ((Ha + Hm) × Wm) mm. The antenna design is based on the concept of a tapered loop antenna, adjusting radius sizes and curvature to control the impedance bandwidth and radiation pattern. In order to ensure that the optimal antenna design can be obtained during antenna design, the objective function will be used to comprehensively evaluate the characteristics of the antenna.

(1) $$\mathrm{Antenna}\;\mathrm{objective}\;\mathrm{function}=\frac{\mathrm{Impedance}\;\mathrm{bandwidth}}{\mathrm{gain}\;\mathrm{standard}\;\mathrm{deviation}\;\times \;\mathrm{group}\;\mathrm{delay}\;\mathrm{standard}\;\mathrm{deviation}}$$

**Figure 5:**
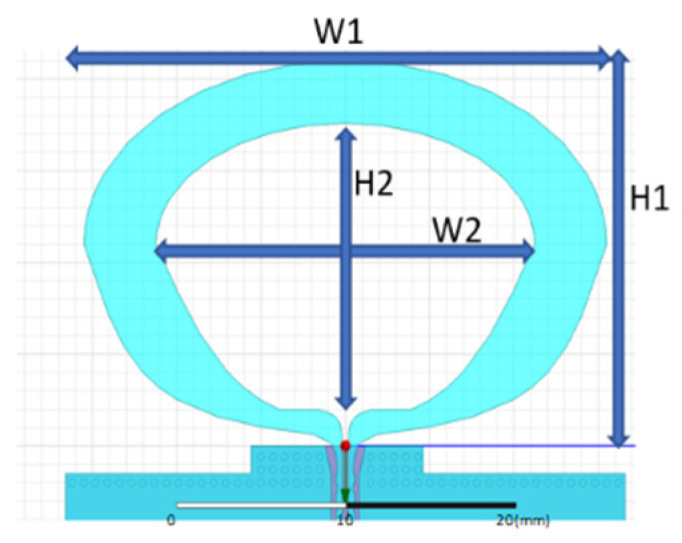
The big-ring structure on the top layer.

**Figure 6:**
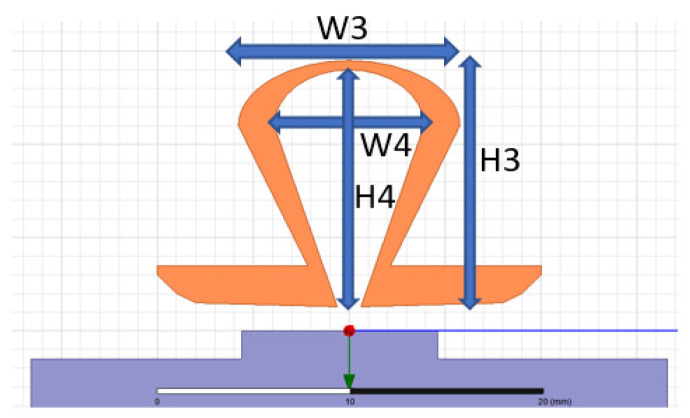
The small-ring structure on the bottom layer.

After optimized design, the antenna integrates a large tapered loop antenna and a small tapered loop antenna, connected at the end by a metal via. The larger tapered loop antenna, positioned on the upper layer, controls low-frequency components, as shown in Figure 5. Meanwhile, the smaller tapered loop antenna on the lower layer manages high-frequency components, as illustrated in Figure 6. Design parameters are detailed in Table 2.

#### 2.1.2. Wireless Precision Positioning Integration Board

In addition to accurate positioning accuracy, positioning systems also need to maintain communication quality, data transmission speed, and expand communication range. To achieve these goals, the key is to develop high-power, high-efficiency radio frequency (RF) front-end modules. In the receiving chain, the Noise Figure (NF) of the first stage plays a vital role [22], so we add a Low-Noise Amplifier (LNA) at the receiving end to suppress noise and amplify the received signal power. In the output link, the output power has a significant impact on the communication distance, so we add a power amplifier (PA) at the output end to increase the output power and extend the communication distance of the positioning system. Finally, single-pole double-throw (SPDT) switches are designed for receive and transmit switching, and LNA, PA, and SPDT switching circuits are integrated into a single-chip design.

**Figure 7:**
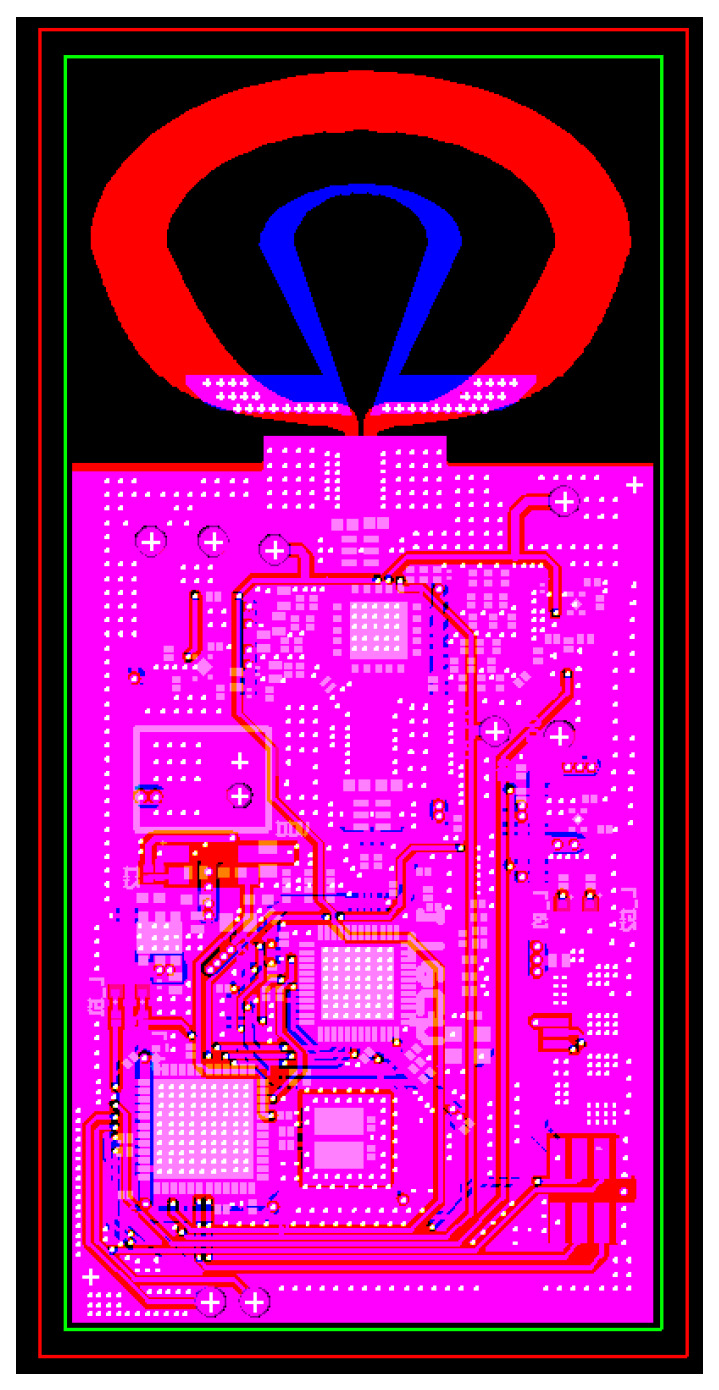
Layout of wireless precision positioning integration board.

**Figure 8:**
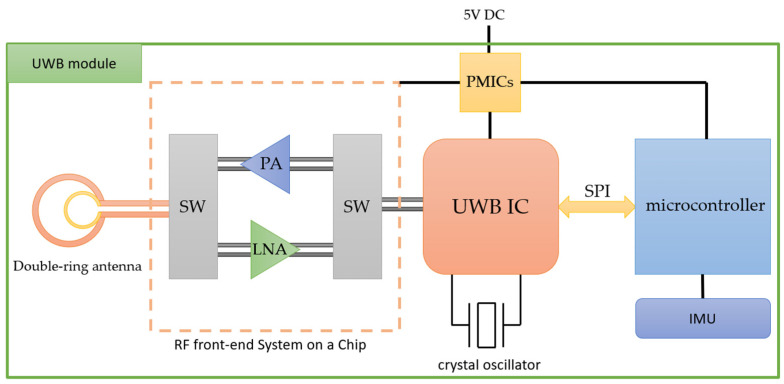
Circuit diagram of UWB module.

As shown in Figure 7, we finally designed an integrated module, including antenna, RF circuit, Serial Peripheral Interface (SPI), Power Management Integrated Circuit (PMIC), IMU, and crystal oscillator. The connections between the antenna, RF front-end module, and individual UWB chips are illustrated in Figure 8 of the circuit diagram. When used with a microcontroller, it can use two-way ranging (TWR) to achieve precise distance calculations [23], making it ideal for real-time location systems (RTLS). This module can configure various parameters through SPI communication through the microcontroller, including data transfer rate, data length, transmission power, UWB channel, preamble and other settings that can be adjusted according to the application scenario. It has a high-precision clock that accurately obtains the timestamp of received packets. Using TWR technology, the TOF of signal transmission can be accurately calculated. In addition, it can capture key parameters such as channel impulse response (CIR), which helps explore the environment through UWB signals and correct ranging errors when non-line-of-sight (NLOS) conditions occur [24,25,26].

### 2.2. Algorithm for Drone Positioning Technology

#### 2.2.1. Gradient Descent Method

**Figure 9:**
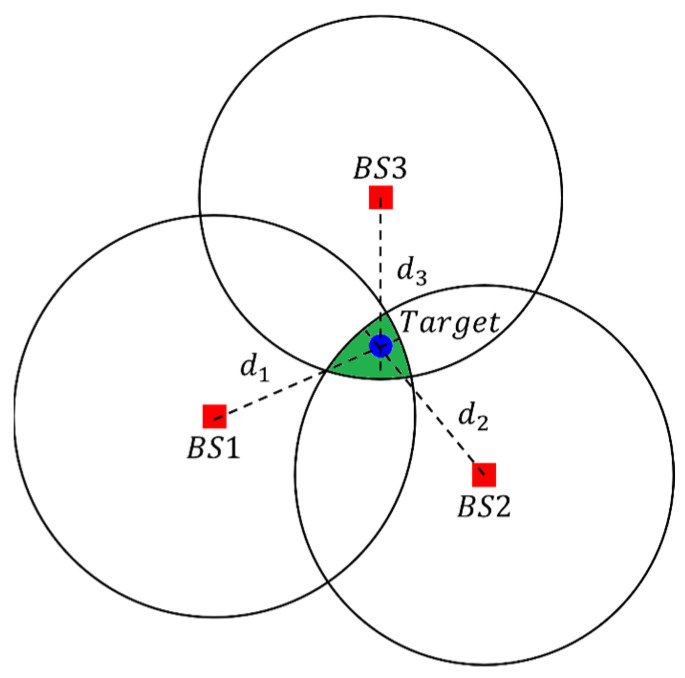
TOA method intersection area.

Under ideal conditions, using Time of Arrival (TOA) enables the intersection at a single point on the map, which represents the target’s position. However, in reality, measurement errors inevitably exist, causing the curves to fail to intersect at a single point and instead form multiple potential interval ranges, as illustrated in Figure 9. Consequently, the direct determination of the true target position becomes unfeasible, necessitating the calculation of an optimized potential position through an iterative process. This project employs the gradient descent method as the approach for optimizing the potential position.

**Figure 10:**
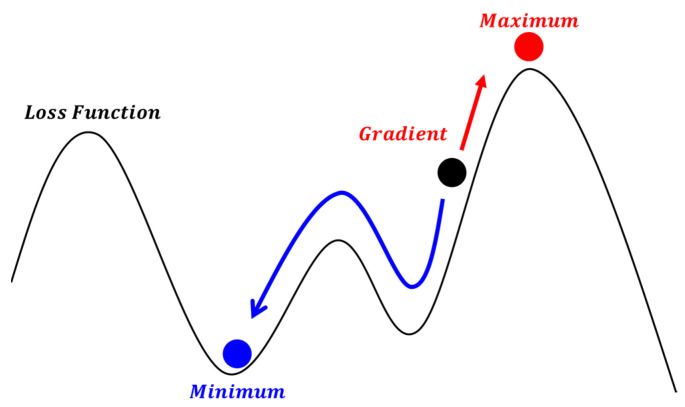
Diagram of gradient descent method.

Gradient descent is a first-order optimization method commonly used to find the maximum or minimum values of a function [27], as illustrated in Figure 10. The distribution of the objective function varies in different regions, implying the possible existence of extremities in each region. To locate these extremities, it is essential to select an initial position and then perform iterative computations along the gradients of the current position until the convergence criteria are met, resulting in a position close to the extremity. The gradient refers to the direction in which the function increases most rapidly at the target position, representing the direction with the highest rate of increase. To obtain a maximum value, one must move along the gradient direction; conversely, to obtain a minimum value, one must move in the opposite direction of the gradient. Generally, the gradient corresponds to the partial derivative of the objective function. To use the gradient descent method for optimizing the target position, it is necessary to define a loss function as the objective function and then apply the gradient descent method to find the position that minimizes the loss function.

Since the TOA measures the distance from the target to the base station, the loss function is defined as follows:

(2) $$\mathrm{Loss}\left(x,\;y\right)=\underset{m}{\sum }{\left(\sqrt{{\left(x-x_{m}\right)}^{2}+{\left(y-y_{m}\right)}^{2}}-r_{m}\right)}^{2}$$

Here, x and y represent the target position to be optimized, m is the station number, ${\;x}_{m}$ and $y_{m}$ are the coordinates of the station, and $r_{m}$ is the measured distance. This loss function aims to minimize the mean square error between the measured distance and the target distance.

The gradient of this loss function with respect to x and y is as follows:

(3) $$\begin{array}{cc} g_{x} & =\frac{\partial }{\partial x}\mathrm{Loss}\left(x,\;y\right)\\ & =2\times \underset{m}{\sum }\frac{\left(\sqrt{{\left(x-x_{m}\right)}^{2}+{\left(y-y_{m}\right)}^{2}}-r_{m}\right)\times \left(x-x_{m}\right)}{\sqrt{{\left(x-x_{m}\right)}^{2}+{\left(y-y_{m}\right)}^{2}}} \end{array}$$

(4) $$\begin{array}{cc} g_{y} & =\frac{\partial }{\partial y}\mathrm{Loss}\left(x,\;y\right)\\ & =2\times \underset{m}{\sum }\frac{\left(\sqrt{{\left(x-x_{m}\right)}^{2}+{\left(y-y_{m}\right)}^{2}}-r_{m}\right)\times \left(y-y_{m}\right)}{\sqrt{{\left(x-x_{m}\right)}^{2}+{\left(y-y_{m}\right)}^{2}}} \end{array}$$

Finally, the following formulas can be used for recursive computation updates of the optimized target positions x and y:

(5) $$x_{k+1}=x_{k}-\eta \times {\frac{\partial }{\partial x}\mathrm{Loss}\left(x,\;y\right)|}_{x=x_{k}}$$

(6) $$y_{k+1}=y_{k}-\eta \times {\frac{\partial }{\partial y}\mathrm{Loss}\left(x,\;y\right)|}_{y=y_{k}}$$

Here, k is the recursive number, η is the learning rate, i.e., the distance moved in each recursion. To achieve the minimum value, it is necessary to move against the gradient, i.e., subtracting the gradient rather than adding it. However, if the learning rate is fixed, it can easily lead to large movement of distances, preventing convergence to the extremum. Therefore, using Adagrad for dynamic updates of the learning rate ensures that the gradient descent method can approach the extremum more closely.

There are generally two convergence conditions, including setting the maximum number of recursions and setting the tolerance for the loss. This study sets the maximum number of recursions to 200 and also considers the distance between the two points before and after each update. If the difference between the distances of the two points before and after the update is less than 1 cm, the gradient descent algorithm stops the computation.

**Figure 11:**
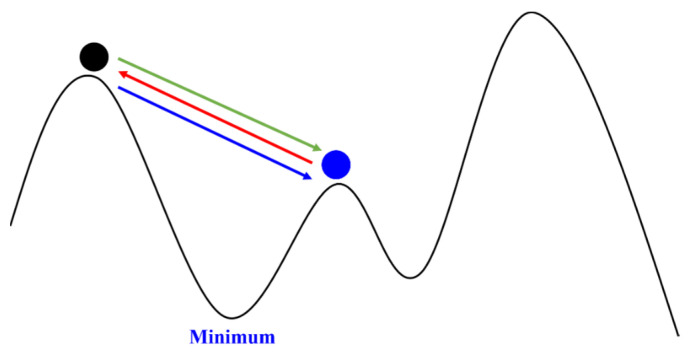
Diagram of learning too fast. The ball will repeatedly move between the blue and black points, unable to reach the minimum value.

As mentioned earlier, the gradient descent method is used to find the extremum of the loss function to optimize the true position of the target. However, in the gradient descent method, if the distance moved each time is fixed, i.e., the learning rate is constant, it can easily lead to the inability to approach the extremum, as illustrated in Figure 11. Since the movement distance is too large each time, it cannot continue to approach the extremum. However, if the learning rate is reduced, it may result in a slow speed, requiring more time for computation. Therefore, it is necessary to adjust the learning rate based on the learning progress. The most common optimization methods are Adagrad and Adam, which will be introduced one by one below.

#### 2.2.2. Adaptive Gradient Method (AdaGrad)

**Figure 12:**
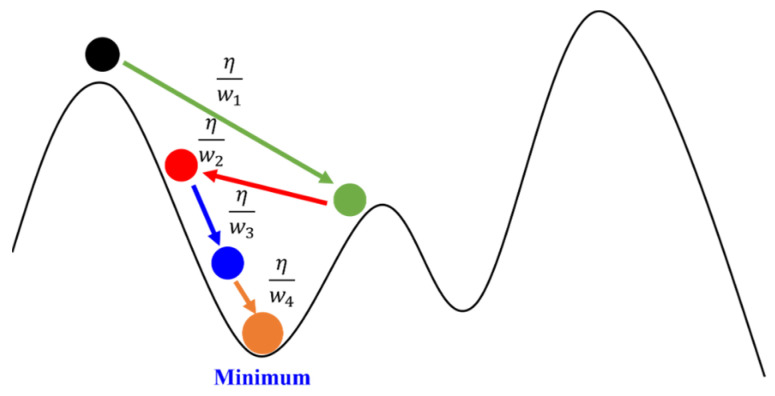
Diagram of Adagrad. The ball gradually decrease to Minimum.

Adagrad is an Adaptive Gradient algorithm, where the distance of travel along the gradient continuously decreases with the number of iterations, as illustrated in Figure 12. Typically, in the gradient descent method, the distance from the extremum is relatively far at the beginning, leading to relatively larger movements. However, as the number of iterations in the gradient descent increases, it gradually approaches the extremum. Therefore, it is necessary to continually reduce the distance of movement to approach the extremum without oscillating back and forth due to excessive movement distance.

(7) $$\eta_{k}=\frac{\eta }{\sqrt{k+1}}$$

The most straightforward adaptive update method is to directly divide the learning rate by the number of iterations, which allows the learning rate to decrease with the number of iterations. However, this alone is still insufficient for effectively approaching the extremum. Therefore, Adagrad considers the previously calculated gradient as a consideration basis and modifies the method of updating the learning rate.

(8) $$\eta_{k}=\frac{\frac{\eta }{\sqrt{k+1}}}{\sigma_{k}}$$

where $\sigma_{k}$ is the root mean square of the previous k gradients.

(9) $$\sigma_{k}=\sqrt{\frac{1}{k+1}\overset{k}{\underset{t=0}{\sum }}{\left(g_{t}\right)}^{2}}$$

Therefore, it can be rewritten as

(10) $$\eta_{k}=\frac{\frac{\eta }{\sqrt{k+1}}}{\sigma_{k}}=\frac{\eta }{\sqrt{\sum_{t=0}^{k}{\left(g_{t}\right)}^{2}}}$$

The gradient descent method can be rewritten as

(11) $$x_{k+1}=x_{k}-\frac{\eta }{\sqrt{\sum_{t=0}^{k}{\left(g_{x,t}\right)}^{2}}}\times g_{x,k}$$

(12) $$y_{k+1}=y_{k}-\frac{\eta }{\sqrt{\sum_{t=0}^{k}{\left(g_{x,t}\right)}^{2}}}\times g_{x,k}$$

In this way, as the number of iterations increases, the distance moved by the gradient descent method will gradually decrease, enabling it to more effectively and accurately approach the correct extremum.

#### 2.2.3. Improved Version of Adagrad (Adam)

**Figure 13:**
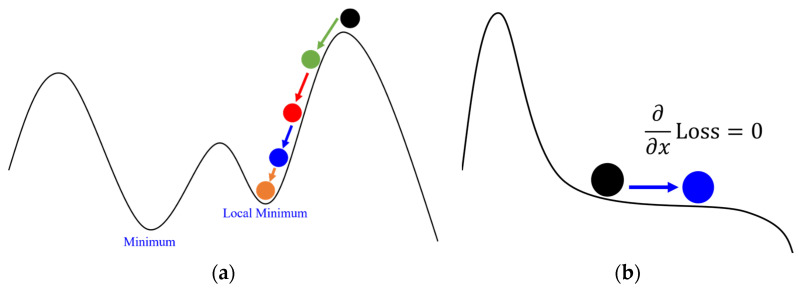
Adagrad do not reach the correct value: (**a**) stuck at local minimum; (**b**) stuck at zero point.

However, because the speed of parameter updates in Adagrad slows down as the number of iterations increases, it eventually becomes unable to approach the true extremum due to the slow movement, getting trapped in a local extremum, as shown in Figure 13a. Alternatively, it may get close to zero, causing the gradient descent method to be unable to approach the extremum, as depicted in Figure 13b. To address the issues of Adagrad getting stuck in local extrema and approaching zero, another common adaptive gradient method, Adam optimization, has emerged.

**Figure 14:**
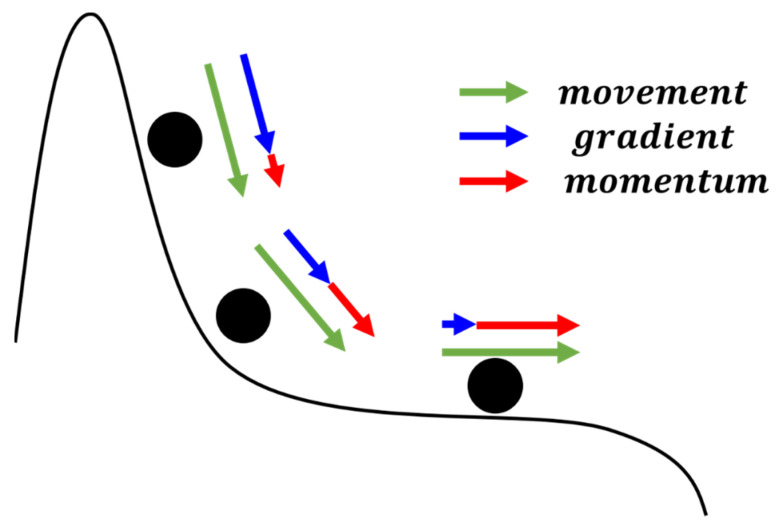
Diagram of Adam optimization method.

Adam is an improved version of Adagrad, mainly incorporating the concept of momentum to simulate real-world physical phenomena, as illustrated in Figure 14. The gradient descent method is akin to a rolling ball. When the ball is rolling downhill, its speed increases until it encounters a peak or valley, where it changes direction, and its speed gradually decreases. If it encounters flat ground, the ball’s speed remains unaffected. Incorporating this physical concept into optimization, Adam considers the previous gradient. If the previous momentum is in the same direction as the current gradient, it means the descent is still downhill, so the update speed should be faster. If the previous momentum is in the opposite direction to the current gradient, it indicates that a peak or valley has been passed, so the update speed should be reduced, gradually turning back. If it encounters a zero point where the gradient is 0, it maintains non-zero momentum, ensuring that the gradient descent method does not stop and can continue moving towards the extremum.

By considering momentum, Adam allows the gradient descent method to cross local extrema with sufficient momentum and has a better chance of escaping local optima. In addition to incorporating the concept of momentum, Adam retains the adaptiveness of Adagrad, considering the square of the gradient as a reference to adjust the learning rate. Therefore, the update formula can be modified as follows:

(13) $$m_{k}=\beta_{1}\times m_{k-1}+\left(1-\beta_{1}\right)\times g_{k}$$

(14) $$v_{k}=\beta_{2}\times v_{k-1}+\left(1-\beta_{2}\right)\times {\left(g_{k}\right)}^{2}$$

(15) $${\hat{m}}_{k}=\frac{m_{k}}{1-{\left(\beta_{1}\right)}^{k}}$$

(16) $${\hat{v}}_{k}=\frac{v_{k}}{1-{\left(\beta_{2}\right)}^{k}}$$

(17) $$w_{k}=w_{k-1}-\eta \times \frac{{\hat{m}}_{k}}{\sqrt{{\hat{v}}_{k}+\epsilon }}$$

where $\beta_{1}$ and $\beta_{2}$ are numbers between 0 and 1, indicating the proportion of the previous momentum to retain. Typically, they are close to 1, commonly set as $\beta_{1}$ = 0.9 and $\beta_{2}$ = 0.999 [28]. ϵ is a very small number primarily used to prevent the denominator from being zero, rendering the expression meaningless.

#### 2.2.4. Kalman Filter Implementation

Since this system is a real-time dynamic positioning system, it is very suitable to use the Kalman filter for filtering [29]. The kinematics in physics are integrated into the Kalman filter. It is assumed that the target obeys the kinematics and other acceleration motion formulas in a very short time. Then, the Kalman filter is implemented in this system.

The kinematic formula of equal acceleration motion is

(18) $$v_{k}=v_{k-1}+a\times t$$

(19) $$p_{k}=p_{k-1}+v_{k-1}\times t+\frac{1}{2}\times a\times t^{2}$$

where $v_{k}$ is the current velocity, a is the acceleration, t is the time difference between two observations, and $p_{k}$ is the current coordinate. Through the kinematic formula, it can be assumed that the hidden state $x_{k}$, the transfer matrix A, the relationship matrix H, and the observation variable $z_{k}$ are respectively

(20) $$x_{k}^{\prime }=\left[\begin{array}{c} \mathrm{position}\\ \mathrm{velocity}\\ \mathrm{acceleration} \end{array}\right]$$

(21) $$A^{\prime }=\left[\begin{array}{ccc} 1 & t & \frac{1}{2}t^{2}\\ 0 & 1 & t\\ 0 & 0 & 1 \end{array}\right]$$

(22) $$H^{\prime }=\left[\begin{array}{ccc} 1 & 0 & 0 \end{array}\right]$$

(23) $$z_{k}^{\prime }=\left[\mathrm{position}\right]$$

The transfer matrix A conforms to the kinematic acceleration motion formula, taking into account both velocity and acceleration. The final observed variable is the observed current position. Finally, the Kalman filter can be rewritten and implemented by performing the same operation for the x and y dimensions respectively.

(24) $$x_{k}=\left[\begin{array}{c} x_{x,k}^{\prime }\\ x_{y,k}^{\prime } \end{array}\right]$$

(25) $$A=\left[\begin{array}{cc} A_{x}^{\prime } & 0\\ 0 & A_{y}^{\prime } \end{array}\right]$$

(26) $$H=\left[\begin{array}{cc} H_{x}^{\prime } & H_{y}^{\prime } \end{array}\right]$$

(27) $$z_{k}=\left[\begin{array}{c} z_{x,k}^{\prime }\\ z_{y,k}^{\prime } \end{array}\right]$$

### 2.3. Flight Control System Design

**Figure 15:**
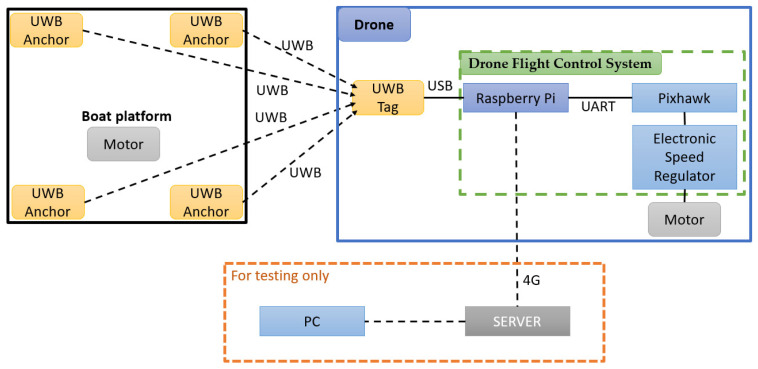
UAV system architecture diagram.

Figure 15 illustrates the system architecture. We constructed a 3 m × 3 m platform on the ship for UAV landing, installing homemade UWB Anchor modules at the four corners of the landing platform.

In UAV systems, the main controller and flight control module are usually separated to prevent insufficient computational performance from causing errors in the flight control system. In this research, the UAV utilizes the Pixhawk flight control module, while the main controller is based on a Raspberry Pi.

Due to the Raspberry Pi having only one set of UART pins, it is connected to the Pixhawk for sending flight control commands. Therefore, the UWB positioning module needs to be connected to the Raspberry Pi’s USB port. This configuration not only allows data transmission but also serves as a power source for the UWB positioning module. In the program, it is handled as a UART transmission.

In practical implementation, it is necessary to connect the Raspberry Pi on the UAV and the ground station computer to the same server. Therefore, the Raspberry Pi can be operated from the ground station using the Secure Shell Protocol (SSH), allowing intervention in the UAV operation at any time in case of emergencies. For testing convenience, this research employs a 4G signal for the Raspberry Pi’s internet connection, and the networking method can be adjusted based on the specific application environment. Thus, the overall architecture ultimately relies on UWB for communication, with the 4G signal only being used during testing.

## 3. Results

### 3.1. Antenna Measurement

#### 3.1.1. Gain and Radiation Pattern

**Figure 16:**
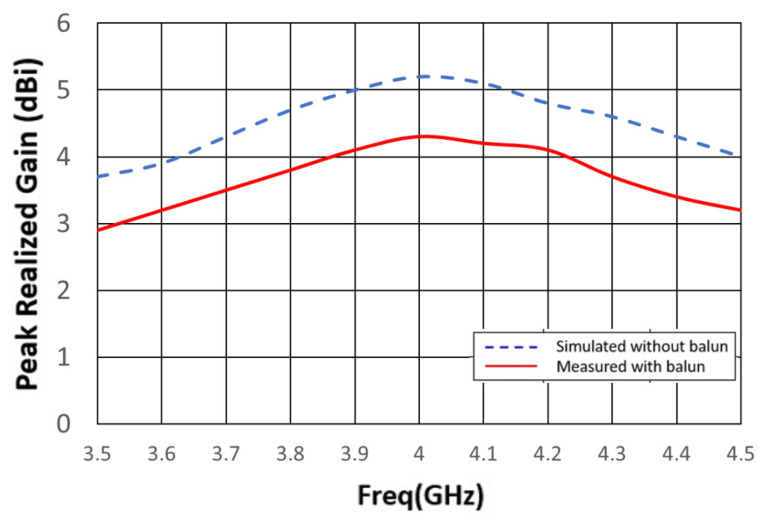
Gain comparison after adding the balun.

**Figure 17:**
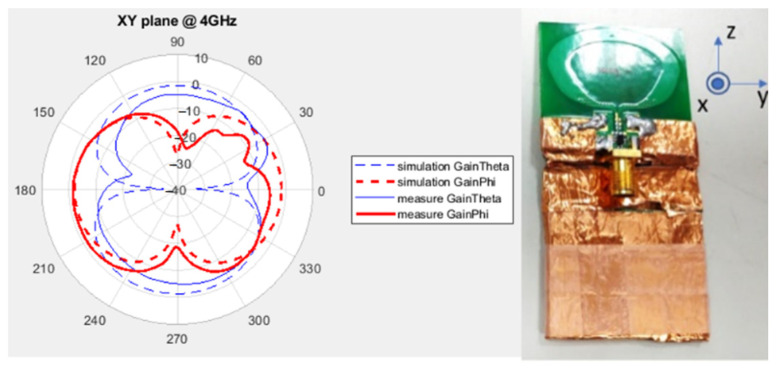
The XY plane radiation pattern at 4 GHz.

The gain and radiation pattern were measured using NSI2000 (NSI-MI Technologies, Suwanee, GA, USA) in an anechoic chamber, and the results are depicted in Figure 16. In simulations, the gain remains approximately 5 dB across the entire operating frequency band. In actual measurements, the gain trend aligns with the simulation results. However, there is a 1 dB gain discrepancy between measurements and simulations attributable to the imperfect characteristics of the balun’s loss. Figure 17 illustrates a comparison between the measured and simulated radiation patterns at 4 GHz. The XZ plane exhibits a more uniform radiation pattern and polarization direction that remains consistent with rotation.

#### 3.1.2. Group Delay

**Figure 18:**
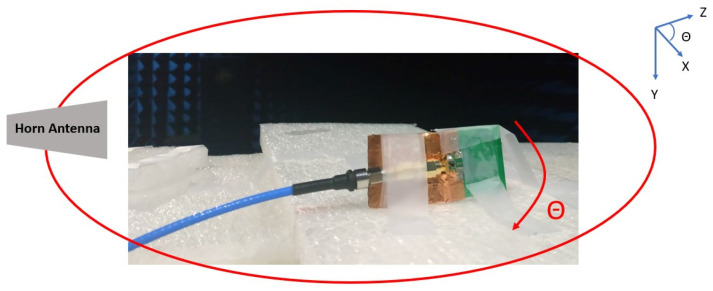
The setup of group delay measurement.

**Figure 19:**
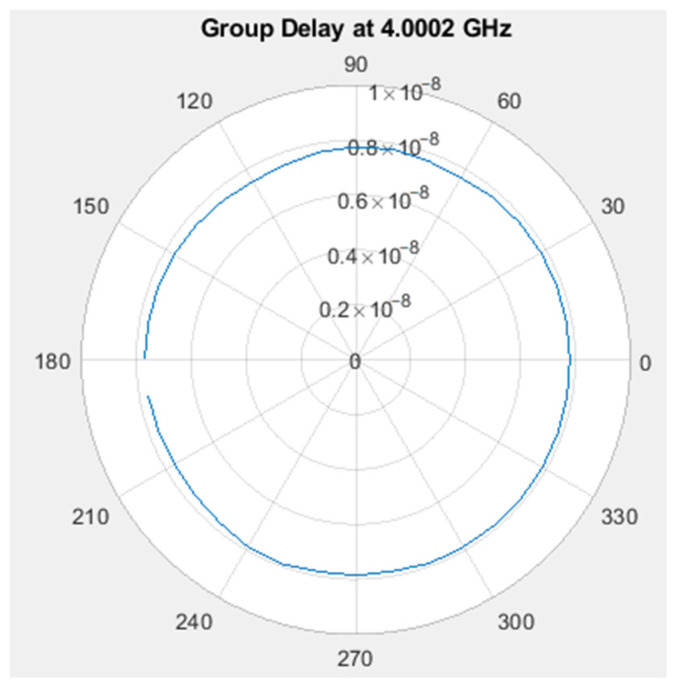
The XZ plane group delay at 4 GHz.

For group delay, our concern is not the absolute value, but rather the changes observed from various angles. Therefore, we utilize the angular standard deviation of the group delay as an indicator of the amount of variation. The calculation method for angular standard deviation is as follows: every 10 degrees corresponds to a point, completing a full rotation around the plane, and calculating the standard deviation of these 36 points. Figure 18 illustrates the group delay measurement setup diagram. The group delay measurement results for the 4 GHz and XZ planes are presented in Figure 19, with an angular standard deviation of 0.11 ns.

To translate the group delay into an actual effect on ranging, one can multiply the group delay by the speed of light, resulting in the distance difference. Taking this antenna as an example for actual measurement, the standard deviation of the group delay angle at 4 GHz is 0.11 ns. This implies that the standard deviation of the ranging distance is only about 3.3 cm.

### 3.2. UWB Module

**Figure 20:**
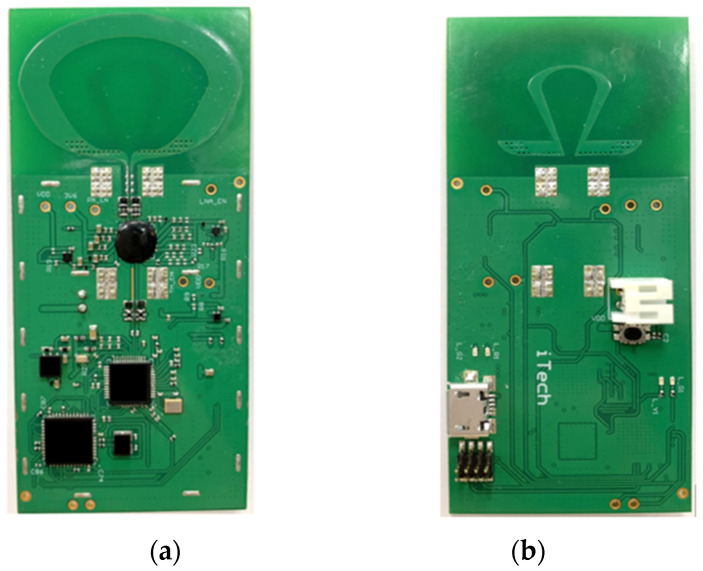
Wireless precision positioning integrated board: (**a**) top view; (**b**) bottom view.

This project independently developed and designed an integrated circuit board that integrates the main chip, transceiver chip, PMIC chip, dual-loop antenna, and more onto a single PCB circuit board. Considering the electrical and radio frequency characteristics, the circuit board was designed using a four-layer FR4 substrate (manufactured by TURBOARD technologies, Taoyuan, Taiwan). The completed physical diagram is depicted in Figure 20a,b.

### 3.3. Positioning Technology Measurement

**Figure 21:**
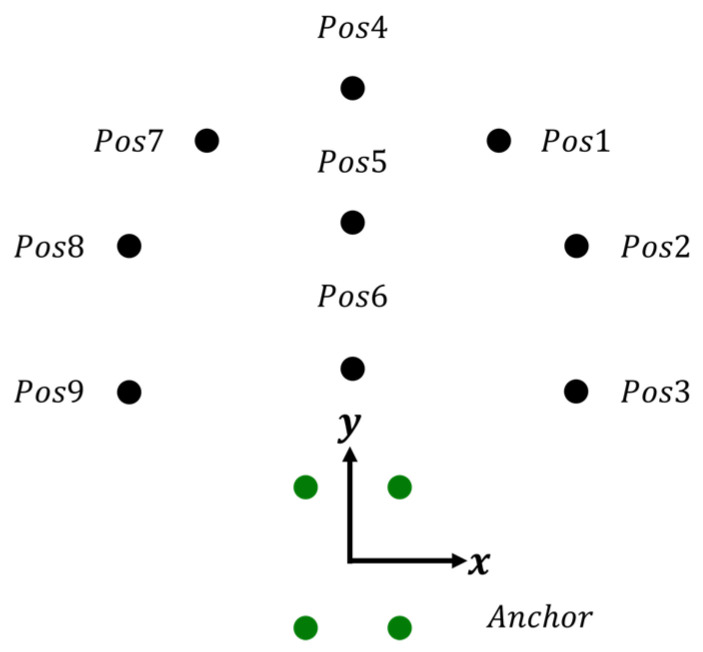
Accuracy test experiment setting diagram. Green dots indicate the placement of the anchor.

To verify the positioning accuracy of the system, experiments were conducted in the laboratory, as illustrated in Figure 21. The devices used in this experiment were the same as those described in the previous chapters. Anchors were placed on stands 1 m above the ground, forming a rectangular shape with dimensions of 60 cm in length and 40 cm in width. Similarly, the tag was set at a height of 1 m above the ground, ensuring that both the tag and anchors were in the same plane. TWR was employed for distance measurement, and the positioning algorithm was carried out using the gradient descent method at various points to measure the positioning results.

**Figure 22:**
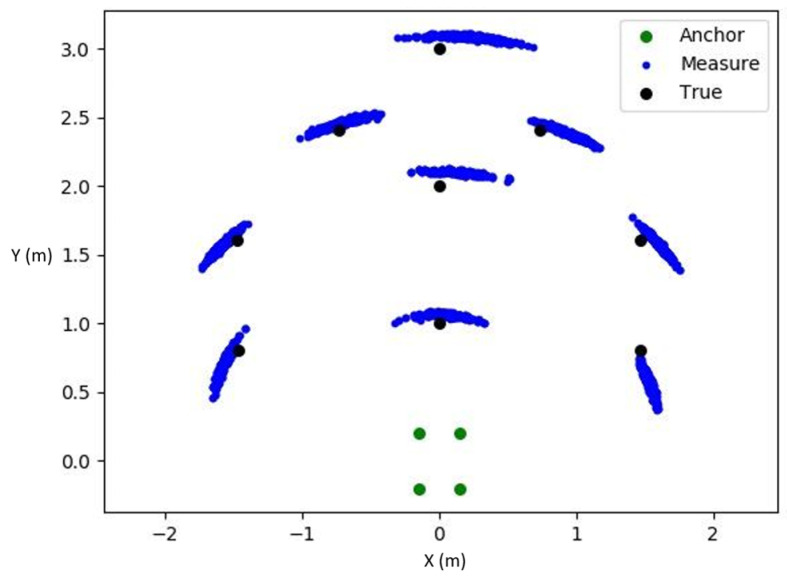
Scatter plot of positioning results.

A total of nine points were selected for measurement in this experiment, and the results are shown in Figure 22. It can be observed that the positioning results at each measurement point exhibited an arc-shaped distribution. The circular center of the arc corresponds to the center of the anchors. This arc-shaped distribution was mainly due to all Tags being located outside the area enclosed by the anchors. Consequently, the four circles formed by the TWR distance positioning method nearly overlapped, creating a narrow arc-shaped region. This led to oscillations along the circumference, resulting in the arc-shaped distribution of the measurement results, indicating a higher accuracy in terms of distance from the center of the anchor but greater oscillation in terms of angle.

**Figure 23:**
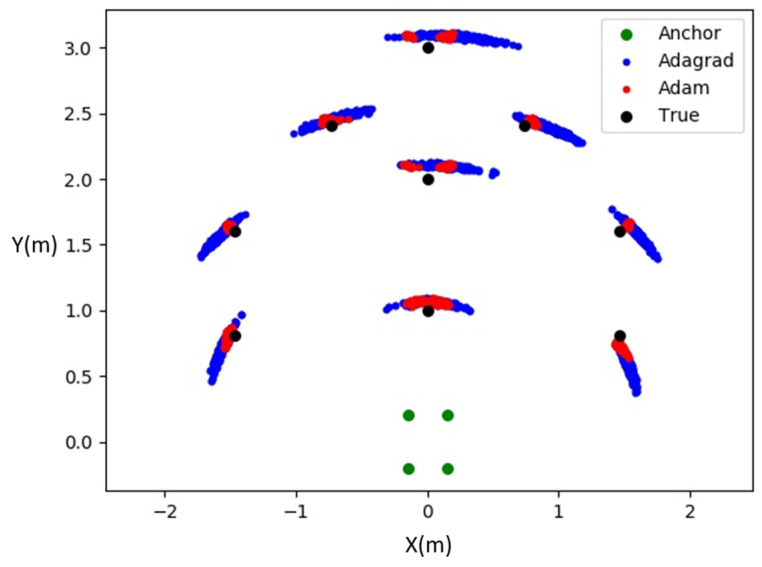
Adagrad and Adam scatter comparison chart.

Furthermore, a comparison was made between the differences in the performance of the Adagrad and Adam methods. Both optimization methods were applied to the same set of measured distance data. The results, as depicted in Figure 23, revealed that the angle oscillation in the scatter plot reduced significantly with the use of Adam, displaying a distribution that was closer to a circular shape. At the same time, the precision of the distance was maintained, demonstrating that the optimization method of Adam could effectively prevent the gradient descent method from approaching local minima or zero points. Instead, it facilitated escaping from local minima and zero points through the use of momentum, resulting in a closer approximation to the true extremum. The comparison of errors between the two methods is presented in Table 3, with the error significantly reduced for Adam, indicating that Adam was more suitable for the system compared to Adagrad.

### 3.4. Drone Control System and Vessel Structure Design

**Figure 24:**
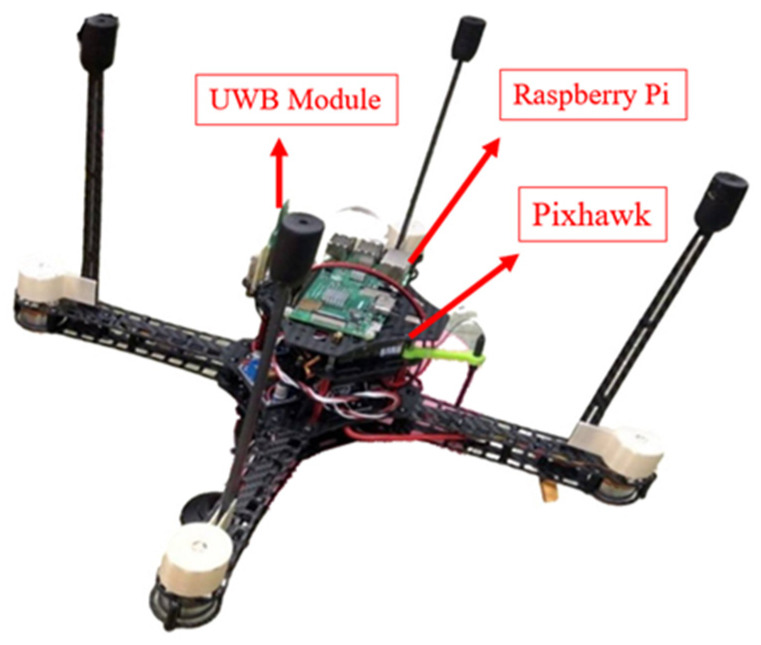
UAV communication system hardware.

Our drone is self-assembled and weighs approximately 750 g, Figure 24 shows the actual assembled UAV communication system hardware, including the UWB tag module, Raspberry Pi, and Pixhawk. To ensure flight safety, they are all installed on the bottom of the UAV. In order to enable the UWB tag module to communicate stably, the UWB tag module is fixed at the bottom and the antenna is perpendicular to the ground. At the same time, the length of the drone’s landing gear is designed to be longer than the UWB tag module antenna to prevent the antenna from touching the ground when landing.

**Figure 25:**
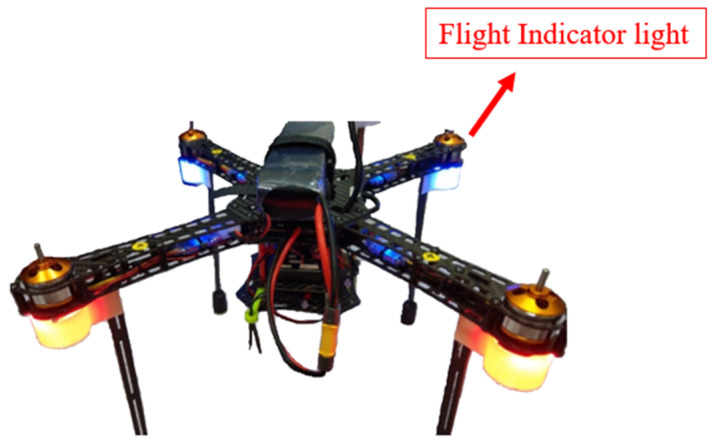
UAV flight indicator light.

When the drone is flying, due to its symmetrical structure, it is difficult for the operator to distinguish the direction visually, so an indicator light is installed to allow the operator to identify the nose of the drone. In Figure 25, the blue indicator light is for the nose of the aircraft, and the orange indicator light is for the tail. A lampshade is installed below the indicator light to make the indicator light easily visible from all angles.

**Figure 26:**
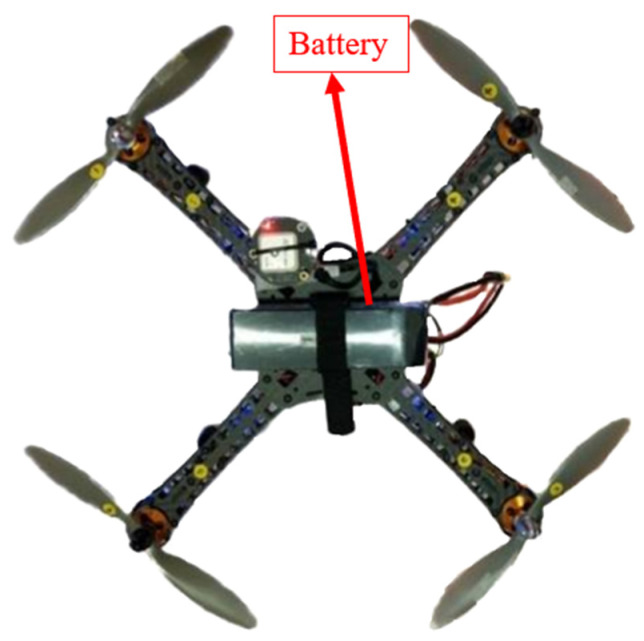
UAV top installation diagram.

Figure 26 shows the installation diagram on the top of the drone. The battery uses a 5800 mAh, 12 V model aircraft battery, which is wrapped around the top of the drone with a fixed strap. According to actual measurements, it can fly for more than 20 min.

**Figure 27:**
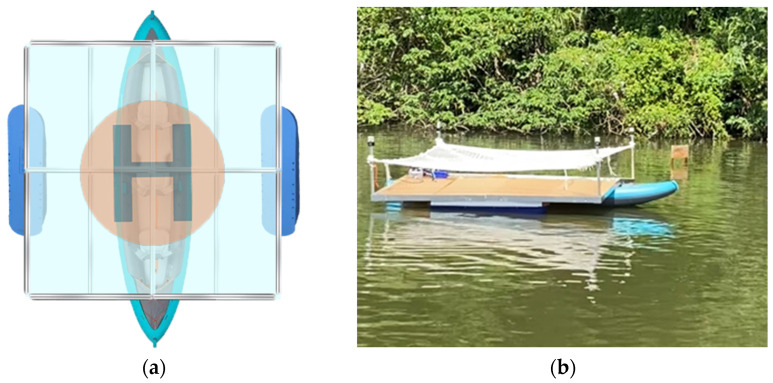
(**a**) Boat structure diagram; (**b**) boat actual operation diagram.

The concept of vessel structure design primarily considers the stability of the platform under the vessel’s motion. To reduce swaying and enhance the load-carrying capacity of the aluminum extrusion structure, the design incorporates an inflatable canoe combined with aluminum extrusions and acrylic panels to form a landing platform. Due to the canoe’s width being only 102 cm, while the installed platform measures 300 × 300 cm, it is prone to a shift in the center of gravity during navigation, leading to swaying and potential misjudgments or submergence of unmanned drones. To avoid this scenario, the design incorporates an auxiliary float on each side of the vessel to increase the platform’s stability, as depicted in Figure 27a, while Figure 27b illustrates the final operational appearance of the vessel’s structure.

### 3.5. Tilt Compensation

As the landing platform is a non-fixed structure prone to tilt and shaking, the reference coordinates of the drone differ from the platform’s basic coordinate system. When the boat tilts, minor positioning errors occur. To rectify these errors, we utilized the accelerometer on the module to compensate for the positioning coordinates.

**Figure 28:**
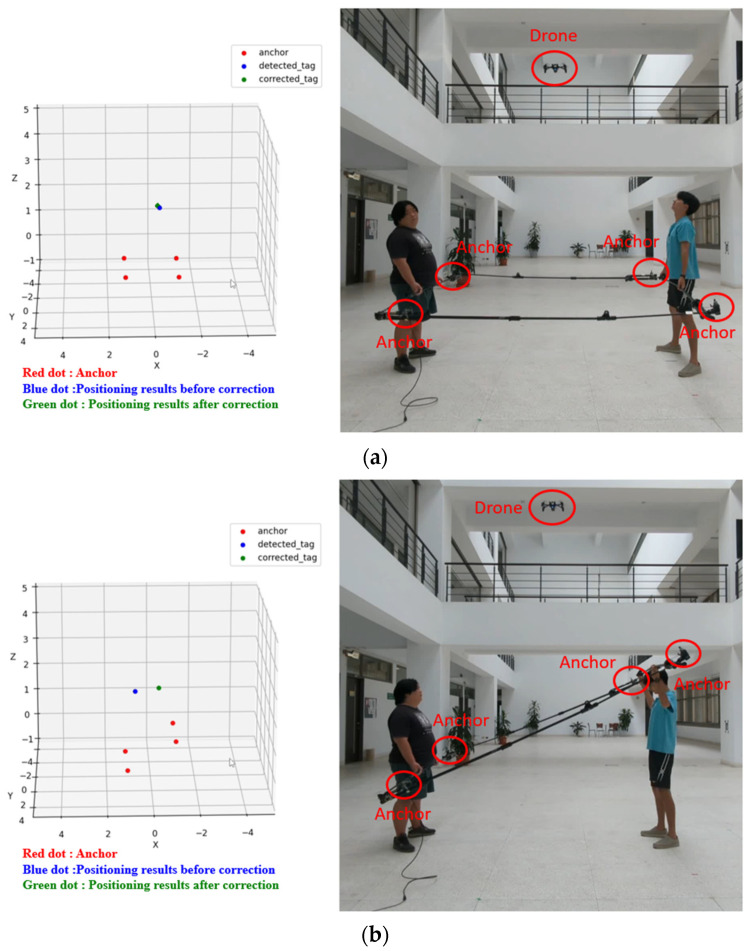

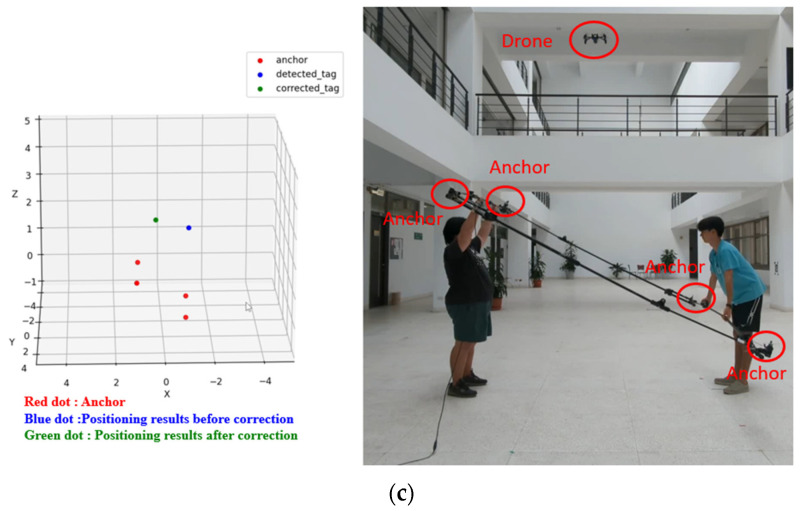
Tilt table positioning compensation test: (**a**) horizontal test; (**b**) left tilt test; (**c**) right tilt test.

To validate the positioning compensation effect of this system on the floating platform, we simulated the floating platform using a 2 m × 2 m square tool. Positioning modules were installed at the four corners, and the tool was operated by two individuals to simulate horizontal and side-to-side tilting. The test results are depicted in Figure 28, where the red dot represents the positioning module, and the blue dot denotes the positioning module. The positioning result before compensation is represented by the red dot, while the green dot represents the positioning result after compensation. Figure 28a illustrates the platform level test, showing a stable anchor point directly above the platform. Figure 28b,c depict left and right tilt tests of the platform, respectively. It is evident that the positioning point is noticeably offset before compensation, while the positioning point after compensation is closer to the correct drone position. This test confirms that the positioning system effectively corrects positioning errors caused by the tilt of the landing platform due to waves.

## 4. Integration and Verification of the Positioning System and Ship-Borne Platform

### 4.1. Static Testing

**Figure 29:**
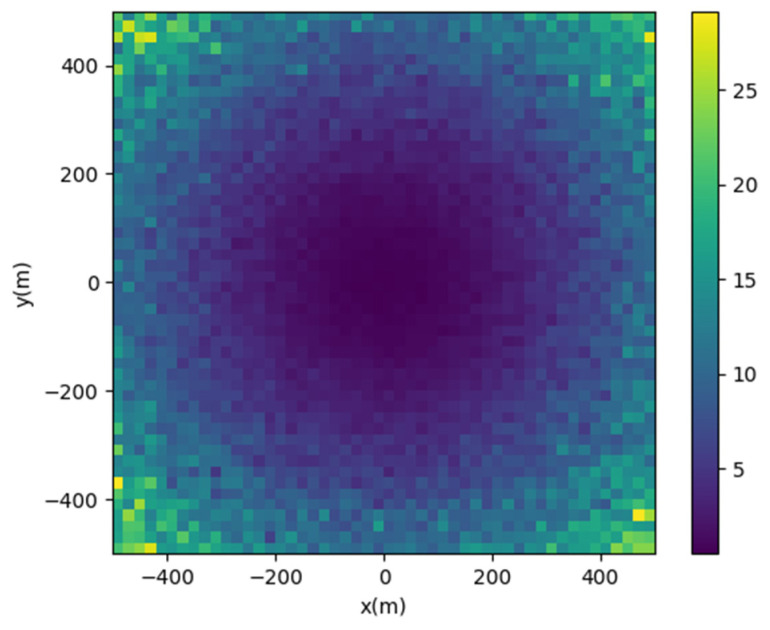
Simulation results of position accuracy.

We simulated the positioning accuracy using the actual statistical standard deviation. However, there are other factors that can affect the positioning accuracy in real environments. We simulated the positioning scenario using a program, setting the landing area as 10 m × 10 m, with anchors placed at the four corners of the landing area. The Tag’s position was set as follows: $x_{T}$~[−500 m, 500 m], $y_{T}$~[−500 m, 500 m], and $z_{T}$ = 100 m, with intervals of 10 m between each point. Each point was simulated 20 times, with each simulation introducing an error equal to the correct distance plus a standard deviation of 0.04 m. This value was derived from actual measurement data statistics. Figure 29 illustrates the simulation results, with colors representing the root mean square error value. Table 4 compares the distance between the tag and the origin, the simulated positioning error, and the actual positioning error.

### 4.2. Dynamic Testing

**Figure 30:**
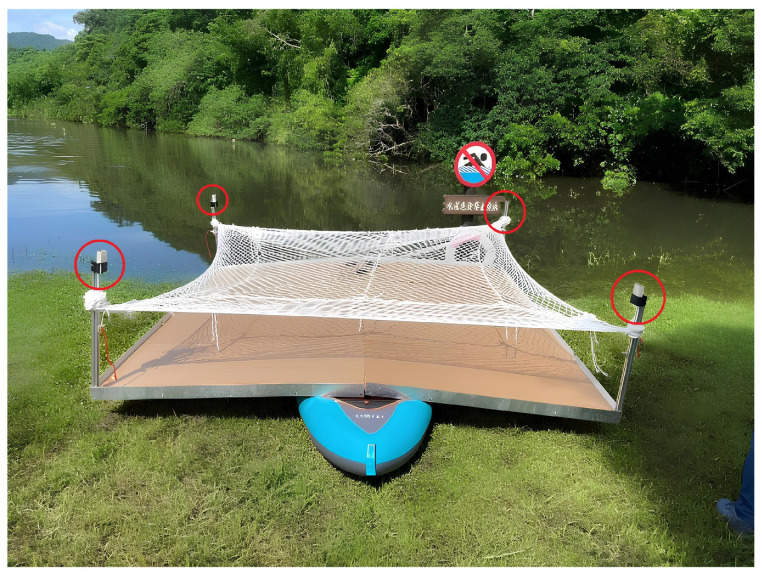
Boat platform (red circle: UWB module anchors).

We installed the UWB module anchors at the four corners of the boat platform, forming a 3 m × 3 m square landing area, as shown in Figure 30. During the actual implementation phase, we will operate the boat on the surface of the lake and employ the module’s positioning system to facilitate the automated landing of the drone on the platform through the utilization of an algorithm. This approach is aimed at achieving a practical demonstration of the automated flying and landing capability.

**Figure 31:**
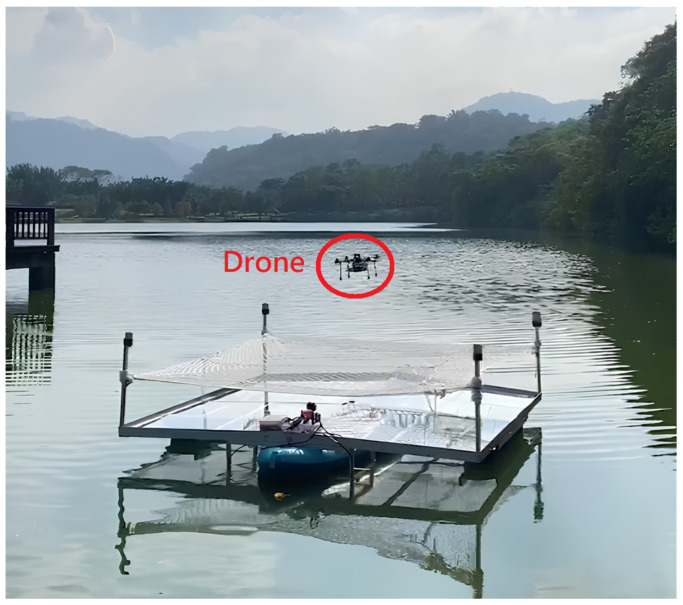
Scenario 1: Drone lands on a stationary boat.

**Figure 32:**
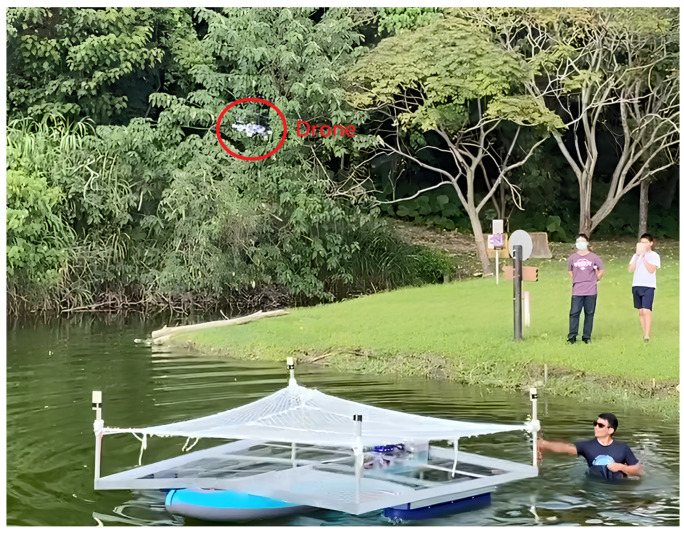
Scenario 2: Drone lands on a swaying boat.

**Figure 33:**
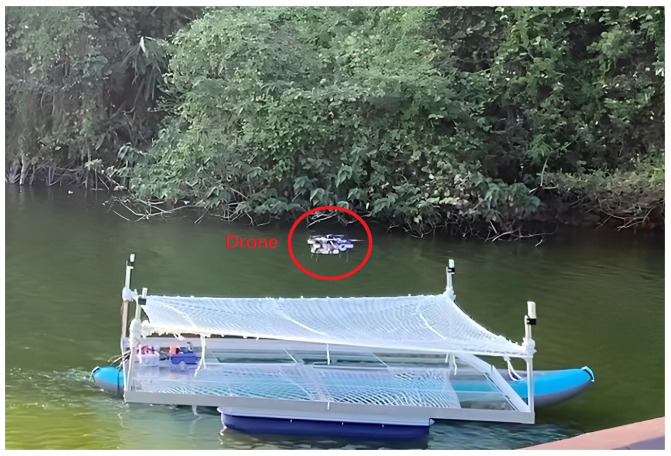
Scenario 3: Drone lands on a moving boat.

We set up three test scenarios. The first scenario is that the drone lands on a stationary boat in the lake, as shown in Figure 31. The second scenario is the drone landing on a stationary but swaying boat in the lake, as shown in Figure 32. The third scenario is that the drone lands on a moving boat, as shown in Figure 33.

## 5. Discussion

**Figure 34:**
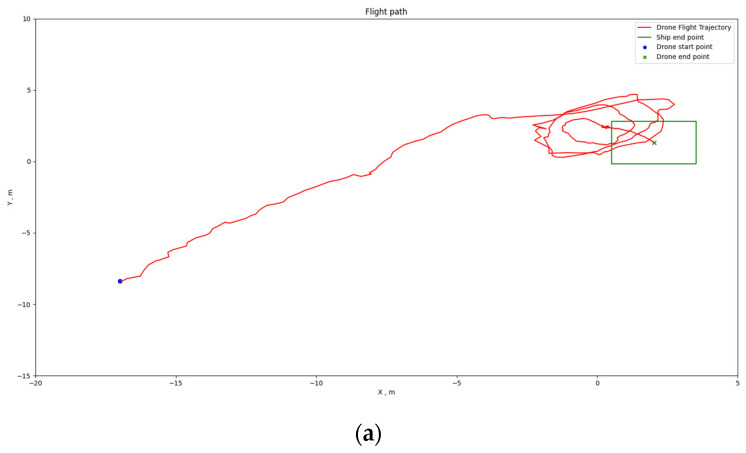

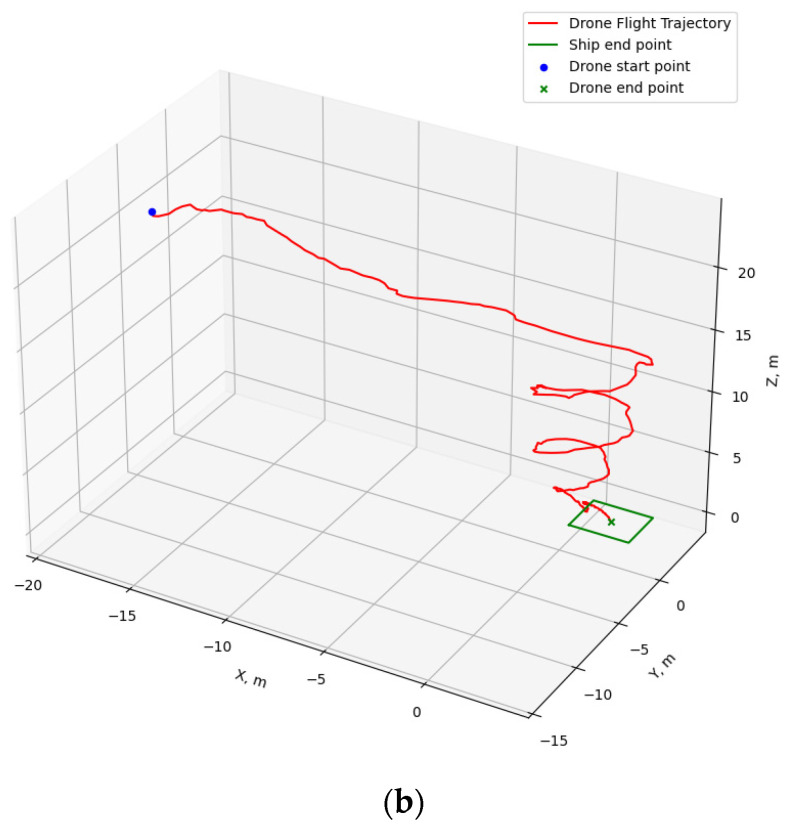
Positioning results of Scenario 1: (**a**) 2D flight path map; (**b**) 3D flight path map.

**Figure 35:**
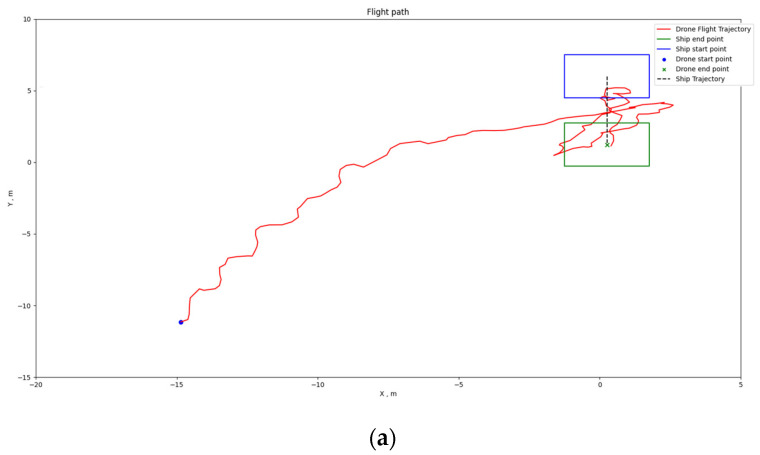

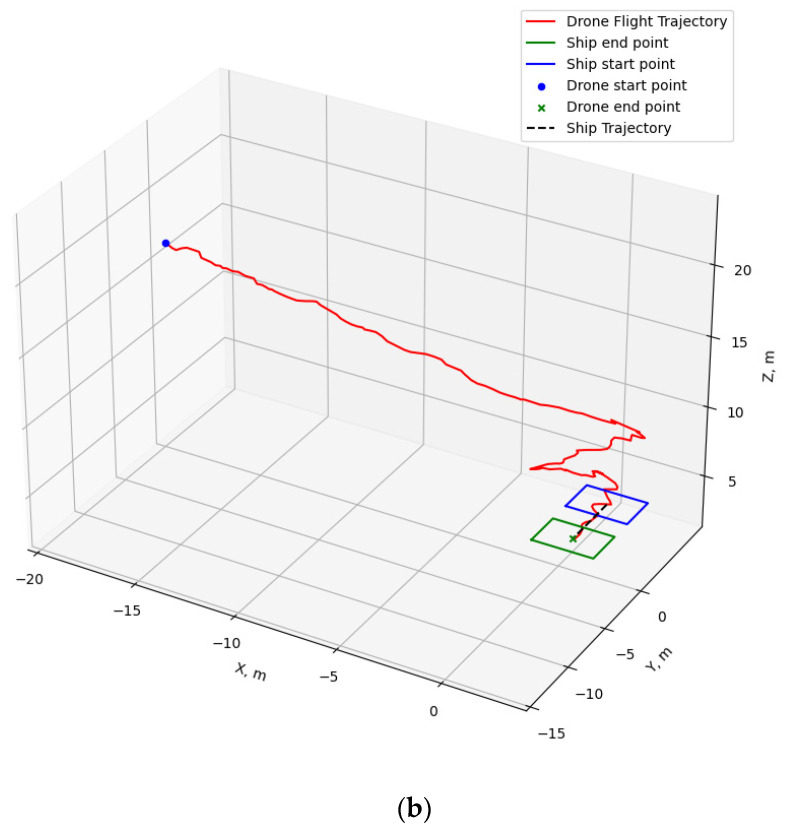
Positioning results of Scenario 2: (**a**) 2D flight path map; (**b**) 3D flight path map.

**Figure 36:**
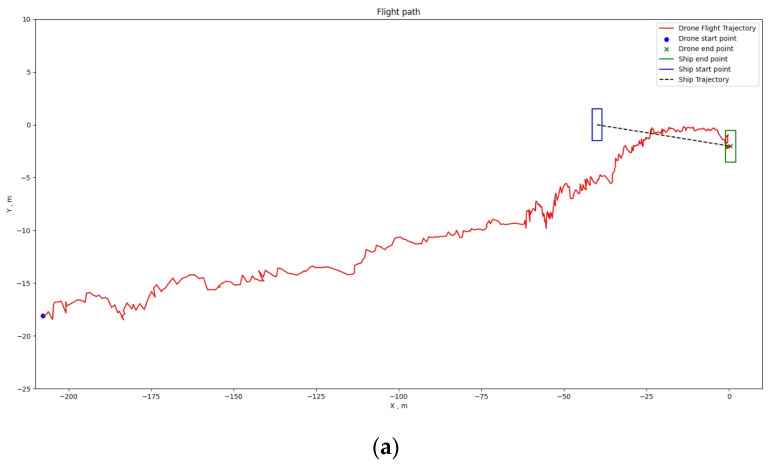

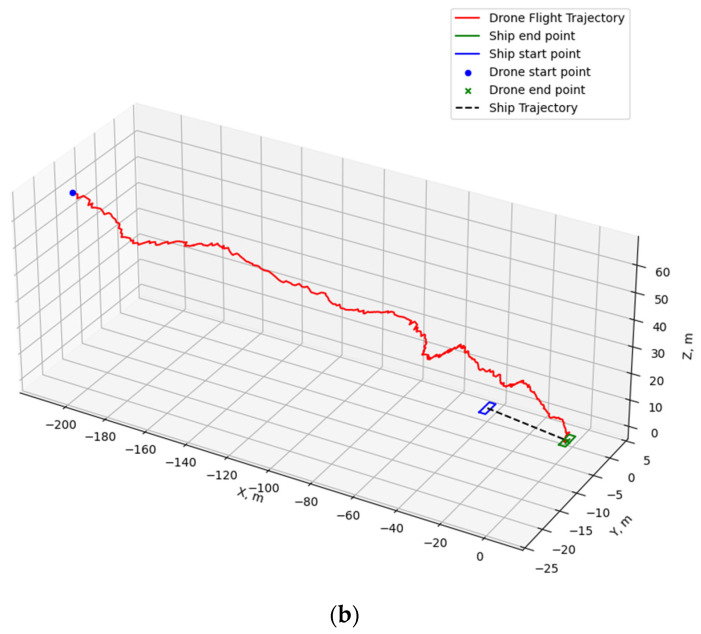
Positioning results of Scenario 3: (**a**) 2D flight path map; (**b**) 3D flight path map.

Figure 34, Figure 35 and Figure 36 depict the positioning outcomes in three distinct scenarios. Our shipborne flying and landing system employ path planning with the goal of minimizing the distance traveled. To ensure the safety of drone flight, we have imposed an upper limit on the descent speed of the drone, ensuring it remains below the horizontal movement speed. This precautionary measure allows for intervention in case of drone loss of control, mitigating the risk of unexpected incidents.

Analyzing the flight trajectory plots reveals that, regardless of the drone’s takeoff position, the shipborne flying and landing system effectively guides the drone’s direction in the XY plane towards the landing point along the shortest path. As the drone approaches the landing point, if its altitude remains significantly above the ship’s platform, the drone adopts a spiraling descent.

Figure 34a illustrates the 2D flight trajectory of the drone when the boat is stationary, while Figure 34b presents the 3D flight trajectory. The drone’s starting point is indicated by the blue circle, descending along the red trajectory line to the green “X” mark. The drone predominantly follows the shortest path towards the ship’s direction in the horizontal plane. However, as the drone approaches the boat horizontally, there is still a 12 m gap in the Z-axis. Consequently, the drone spirals down to the designated point, ensuring a safe and precise landing on the platform.

Figure 35a,b showcase the 2D and 3D flight trajectories of the drone when the boat is swaying. Despite slight lateral movements caused by the significant sway of the boat, the drone generally follows the shortest path in the horizontal direction. This demonstrates the effectiveness of our inclination correction in assisting the drone’s landing. Additionally, due to the sway of the ship, there is a deviation between the ship’s final and initial positions. When the drone approaches directly above the boat, there is approximately a 7 m height difference. Therefore, the drone gradually corrects its position through a spiraling descent and lands precisely at the platform center.

Figure 36a,b present the 2D and 3D flight trajectories of the drone when the boat is in motion. The trajectory plots show that the drone adjusts its path according to the boat’s movement. The blue box outlines the boat’s initial position, and the green box represents the boat’s position when the drone lands. In the 3D plot, it is evident that when the drone’s horizontal position is directly above the boat and the height difference with the boat is minimal. Consequently, the drone does not spiral down and accurately lands at the platform center while the boat is in motion.

These results highlight the effectiveness of our shipborne flying and landing system in safely and precisely guiding drones to their designated landing points. The system successfully adapts its trajectory planning to various operational conditions, including ship motion and swaying, ensuring optimal performance.

## 6. Conclusions

In summary, this study presents a wireless precise positioning system for shipboard aircraft that operates without GPS assistance. Through the self-developed UWB module, positioning algorithm, and flight control system, it is ensured that the UAV can automatically land on the ship without using GPS signals.

Actual measurements demonstrate a more than sixfold increase in UWB communication distance compared to previous studies [16], extending the range to 200 m. The introduction of a self-adaptive gradient method and tilt compensation using IMU data enhances efficiency in real-world maritime scenarios and addresses challenges arising from the instability of the landing platform. Our system eliminates the requirement for a GPS module, given that the IMU is seamlessly integrated into the UWB module, and communication is exclusively dependent on UWB signals. Therefore, the establishment of additional communication channels is unnecessary, and the automatic landing process of the UAV can be entirely dependent on UWB communication. Empirical results validate the system’s performance in both static and dynamic scenarios. The shipborne flight and landing system successfully navigates the UAV to precise landing points, demonstrating robust capabilities even on dynamically moving platforms.

## Figures and Tables

**Table 1:** Positioning accuracy comparison table.

Positioning Technology	Advantages	Disadvantages
Ultrasound	- Precise distance measurement. - Not easily affected by environmental light.	- Unstable detection results due to a small target size.
Infrared	- Offers precise voltage changes in photon sensors.	- Highly influenced by materials and wavelengths.
WiFi	- Utilizes signal strength or channel information. - Existing WiFi devices serve as base stations.	- Requires modeling; accuracy limited to the meter range.
Bluetooth	- Calculates distance using Received Signal Strength. - Lower power consumption and costs than WiFi.	- Less precise than TOF, not commonly used for accurate positioning.
Laser	- Uses TOF for accurate distance measurement. - Offers high-precision distance and flight time measurements.	- Affected by weather, limiting suitability for specific climates.
Cameras	- Common devices for analyzing images, determining depth and angle of targets. - Capable of target image recognition.	- Limited by lighting conditions and coverage angles.
UWB	- Uses pulse signals for TOF measurements, ensuring high-precision positioning unaffected by environmental light.	- Higher hardware requirements, potentially increasing costs.

**Table 2:** Antenna geometry parameters.

Parameter	Size (mm)	Parameter	Size (mm)
Ha	21	H2	17.6
Wa	31	W2	22.4
Hm	50	H3	13.2
Wm	33.6	W3	11.6
H1	21	H4	12.7
W1	31	W4	7.8

**Table 3:** Comparison of errors between the two methods.

MSE(m)	Adagrad	Adam
Pos1	0.237	0.073
Pos2	0.155	0.077
Pos3	0.255	0.089
Pos4	0.282	0.186
Pos5	0.220	0.187
Pos6	0.141	0.114
Pos7	0.131	0.053
Pos8	0.143	0.059
Pos9	0.175	0.059

**Table 4:** Positioning accuracy comparison table.

Distance (m)	Root Mean Square Error of Simulated Positioning (m)
10	0.11
50	0.53
100	0.99
200	2.18
300	4.16

## Data Availability

The data are not publicly available due to privacy concerns.
